# Levels of undernutrition and associated factors among adults receiving highly active anti-retroviral therapy in health institutions in Bench Maji Zone, Southwest Ethiopia in 2018

**DOI:** 10.3389/fnut.2022.814494

**Published:** 2022-08-09

**Authors:** Tilahun Mekonnen Regassa, Tesfaye Abera Gudeta

**Affiliations:** Department of Nursing, Mizan Tepi University, Mizan Aman, Ethiopia

**Keywords:** undernutrition, associated factors, HAART, MTU, Ethiopia

## Abstract

**Background:**

Nutritional issues are common in people with Human Immune Virus (HIV). At some point, almost everyone living with HIV faces challenges in maintaining good nutrition. There is insufficient evidence-based information on undernutrition in adults living with HIV on Highly Active Anti-Retroviral Therapy.

**Objective:**

To assess the magnitude of undernutrition and associated factors among patients receiving Highly Active Anti-Retroviral Therapy in health facilities in the Bench Maji Zone, southwest Ethiopia in 2018.

**Methods:**

A facility-based cross-sectional study design was employed from 1 May to 30 June 2018 on 1,007 study subjects and the participants were selected by using a consecutive sampling technique. Five health facilities were selected through a simple random sampling technique. Data were entered into Epi Data Statistical software version 3.1 and analyzed using Statistical Package for Social Sciences (SPSS) software version 21.0. Logistic regression analysis was used to identify factors associated with undernutrition in adults receiving ART. Odds ratios with 95 % confidence intervals were used to examine associations between dependent and independent variables.

**Result:**

Of the total 1,007 study subjects, 961 participated in the study. More than half of the participants were female (61.2%), 57.2% were married and 42.9% were in the 30–39 years age group. In this study, the level of undernutrition among patients on antiretroviral therapy was 16%. Age ≥50 [AOR 2.5, 95% CI (1.1–5.6)], being single [AOR 2.2, 95% CI (1.4–3.7)], developing gastrointestinal symptoms [AOR 2.6, 95% CI (1.5–4.4)] and in WHO-defined clinical stages III and IV of HIV/AIDS [AOR 2.8, 95% CI (1.3–6.0)] were found to have a statistically significant association with undernutrition.

**Conclusion:**

Significant numbers of people on highly active antiretroviral therapy in the study area were undernourished. This demonstrated that HIV/AIDs and its treatment directly or indirectly impacted the nutritional status of the patients, who need a critical follow-up from health workers. Age, marital status, progressing to WHO-defined clinical stage of disease, and development of gastrointestinal symptoms were identified as factors that contribute to undernutrition among patients on highly active antiretroviral therapy. The health care workers and experts working at the ART clinic should focus on patient counseling regarding the early prevention, detection, and treatment of opportunistic infections. Early health-seeking behaviors before the AIDs stage and critical follow-up are the first actions to identify undernutrition.

## Introduction

Malnutrition, specifically undernutrition, and HIV are highly interrelated. Malnutrition contributes to HIV infection and shortens the progression time to the AIDS stage, and HIV infection escalates the risk of becoming malnourished as it reduces appetite and absorption, develops metabolic fluctuations, chronic infections, anorexia, diarrhea, fever, nausea, vomiting, thrush, and anemia. Moreover, people living with HIV cope with difficulties in maintaining good nutrition, because of the infection itself and the effects of Highly Active Anti-Retrovirus Therapy (HAART). The virus causes inflammation of the intestine, leading to difficulty in the absorption of nutrients and medicines, which can result in undernutrition and deficiencies of minerals and vitamins. Nutrition is important even if the individuals start HAART to maintain health and build the immune system of the body (1, 2).

Globally, approximately 36 million people are living with HIV/AIDS, 25 million of them in Africa and the epidemic is common in populations struggling with malnutrition (2, 3). The increase in HIV/AIDS is driven by malnutrition and conversely contributes to malnutrition. Integrating nutrition into the HIV/AIDS treatment protocol and preventing the infection are crucial actions in combating these challenges (4).

The prevalence of both HIV/AIDS and malnutrition is high in several parts of the world, including sub-Saharan Africa. Their consequences are interrelated and aggravate one another. Both can individually cause impaired immune systems and increase the risk of opportunistic infections, diarrhea, fever, loss of appetite, mal-absorption, and weight loss. HIV affects the nutritional status and people living with HIV who have a healthy diet can tolerate HAART, maintain a healthy weight, and can feel better generally (5, 6).

In Ethiopia, HIV is an epidemic. The data extracted from the EDHS 2016 survey shows that the overall national prevalence of HIV infection in Ethiopia is 0.9%, of which 1.2% is among women and 0.6% among men (7, 8). The studies conducted in Ethiopia demonstrate that the proportion of patients on ART with BMI <18.5 kg/ m^2^ varied with region and year; for instance, 43, 31.2, 27, 25.2, 23.2, 26.6, 25.5, and 30% in Hossana (9), Nekemte (10), Butajira (11), Dembia district (12), Wolaita Sodo (13), Bahir Dar (14) and East Harage Zone respectively (15). The recently published studies in other regions of Ethiopia indicated that the prevalence of undernutrition was 18.3, 34, 34.9, and 42.9% in Asella, Jimma, western Ethiopia, and the Eastern Zone of Tigray respectively (16–19). According to literature, commonly indicated predictors of undernutrition are: age, sex, marital status, educational status, household income, residence, source of drinking water, WHO clinical stage, opportunistic infections, CD4 cell count, gastrointestinal symptoms, duration of HAART, and so on (20–24).

The current level of undernutrition among patients on HAART in the study area is unknown and its associated factors and context are varied. Thus, this study aimed to assess undernutrition and its associated factors among patients receiving HAART in Bench Maji Zone health facilities.

## Methods and materials

### Study area and period

This study was conducted in the Bench Maji Zone from 1 May to 30 June 2018. This Zone is located in the Ethiopian Southern Nations, Nationalities, and Peoples' Region (SNNPR) and is 565 km away from the capital city. The administrative center of the Zone is Mizan Aman town and consists of one city administration, and 10 Woredas (local administrative units larger than a village). In 2018, there were 2,499 patients on HAART in 12 health facilities in the Zone where this therapy is being provided. In the Bench Maji Zone, there is one teaching hospital and 39 health centers.

### Study design

A facility-based cross-sectional study design with quantitative methods of data collection was employed to assess undernutrition and associated factors among patients accessing the HAART clinic.

### Population

All patients receiving HAART the in health facilities of Bench Maji Zone were the source population and all sampled participants on HAART from selected health institutions were study subjects.

All patients who were 18 years and above, and those who started HAART in selected health facilities were included in the study. Patients who had kyphoscoliosis (for height measurement), were critically ill and unable to communicate, had HIV but were not yet on ART, and pregnant women were excluded from the study.

### Sample size determination and sampling technique

The sample size was calculated using a single population proportion sample size calculation formula with assumptions of a 3% margin of error and 95% confidence intervals, α = 0.05 (level of significance), and *P* = 31.2% as the proportion of undernutrition among people on ART (9). After adding a 10% non-response rate, the final sample size was 1,007. Of the health facilities that provided the ART service, the following five were selected by the simple random sampling (SRS) technique of lottery method: Mizan Tepi Teaching University Hospital, Sheko Health Center, Mizan Health Center, Biftu Health Center, and Gizmeret Health Center. Based on the source of the population, the sample size was proportionally allocated to each health facility. The study participants were interviewed consecutively until the sample size was reached.

### Operational definition and terms

**Undernutrition** is the outcome variable in this study and is operationalized as the BMI of clients on HAART which is <18.5, after calculation by using the formula: **BMI** **=** **Weight in kgs/ Height in meters**^**2**^ (5). The severity of undernutrition was determined through the following parameters: **severe** (BMI ≤ 16), **moderate (BMI 16–16.99), and mild (BMI 17–18.49)**.

**Dietary diversity** was computed and divided into two categories. From the nine food items score, if it was < 4 (≤4) it was taken as a **low dietary diversity** score, and if was > 5 (≥5) it was determined as a **high dietary diversity** score.

**Good adherence:** A good adherence was when the average adherence was >95% (the participant missed ≤ 2 doses of 30 doses or ≤ 3 doses of 60 doses). **Fair adherence**: If the average adherence is 85%−94% (missed 3–5 doses of 30 doses or 3–9 doses of 60 doses). **Poor adherence:** If the average adherence is <85% (missed ≥ 6 doses from 30 doses or >9 doses of 60 doses) (10).

**Food frequency**: The pattern of food consumption of participants each day. It was classified as **poor** if < 3 and **good** if ≥ 3 per day.

**Woredas-** the term Woreda is a lower local or small geographical administrative region/structure of the government. The government structure /hierarchy in Ethiopia is Nation-Region-Zone-Woreda-Kebele (village).

### Data collection technique and tools

The data were collected using a pre-tested structured questionnaire, which was adopted after reviewing different studies (10–13, 23, 24). Dietary diversity was calculated using the questionnaires adopted from FAO guidelines (25); a food diversity questionnaire was used to obtain information about the food consumption pattern of the participants and a total dietary diversity score was calculated from a 24 h recalled list of food items consumed the previous day. This list of food items was grouped into nine food groups and if the food diversity score was four or less it was taken as a low dietary diversity score (13, 25). Food frequency was the score of how often the individual ate per day and if it was less than three times per day, it was scored as poor food frequency (13). All medical factors were assessed according to the ART follow-up on the registration book. The face-to-face validity was checked by experts. The questions and responses were grouped and arranged according to the particular objectives they addressed.

The body weight of the participants was measured using the standard beam balance in the medical setup and recorded to the nearest 0.1 kg; during measurement, participants were lightly dressed and shoes removed. Women wearing accessories such as scarves were asked to remove them during body weight measurements. Similarly, height measurements were recorded after the participants removed their shoes. They had to stand erect, look straight, with feet together and knees straight. During this measurement, the heels, buttocks, shoulder blades, and the back of the head were adjusted to touch against the wall and the measurements were recorded to the nearest 0.5 cm (11).

### Data collectors and data collection procedure

Eight clinical nurses who work in ART clinics were recruited purposively as data collectors. Two supervisors who had MSc in Health were recruited. Data were collected through face-to-face interviews using a pre-tested structured questionnaire and a patient ART registration book.

### Quality control measures

The quality of the data was assured by using pre-tested questionnaires. Before the actual data collection, a pilot test was conducted on 5% of the total sample size, and the participants were selected from health facilities that were not included in the analysis of the actual study. Based on these initial findings, necessary amendments were made to the tool. Data collectors were trained intensively for 2 days on the study instrument, and they worked under close supervision of the supervisors to ensure adherence to correct data collection procedures. The investigators periodically reviewed the filled questionnaires during the data collection process for completeness, after which, the data were carefully entered and cleaned before beginning the analysis.

### Data processing and analysis

EPI data statistical software version 3.1 was used for data entry and statistical package for social sciences (SPSS) software version 21.0 was used for the analysis. After organizing and cleaning the data, frequencies and percentages were calculated for all variables that were related to the objectives of the study. Variables with *P*-value ≤ 0.25 in the binary logistic regression analysis were entered into the multivariable logistic regression analysis to control confounds. Odds ratios with 95% confidence intervals were used to examine associations between dependent and independent variables. *P*-value ≤ 0.05 was considered significant. Finally, the result was presented using tables, and charts.

### Ethical considerations

Ethical clearance from the Mizan Tepi University research committee, written permission from respective authorities, and verbal consent of the respondents were obtained before data collection. To elicit co-operation, respondents were reassured about the confidentiality of their responses. They were also appraised of their voluntary participation, confidentiality, anonymity, and the right to take part or terminate at any time they wanted. The research assistants were trained by the principal investigators on how to maintain the confidentiality and anonymity of the respondents' responses in all aspects.

## Results

### Socio-demographic characteristics of the study participants

Out of the total 1,007 respondents, 961 participated in the study which gives a 95.4% of response rate. More than half of the study participants (*n* = 588, 61.2%) were women, 412 (42.9%) were in the 30–39 age group, 550 (57.2%) were married, and a majority of them (*n* = 681, 70.9%) were urban residents. More than half of the respondents (*n* = 555, 57.8%) were orthodox followers by religion, 411(42.8%) had attended primary education, most of them 905 (94.2%) had ≤5 family size, and roughly half (*n* = 503, 52.3%) lived with their spouse and children. Most of the participants (*n* = 578, 60.1%) used tap water and half of them got <1000 ETB (19.2 USD) average family monthly income (Table 1).

**Table 1:** Socio-demographic characteristics of the study participants at Bench Maji Zone, southwest, Ethiopia, 2018.

**Variable**	**Category**	**Frequency**	**Percent**
		**(*n* = 961)**	**(%)**
Age	18–29	320	33.3
	30–39	412	42.9
	40–49	179	18.6
	≥50	50	5.2
Sex	Male	372	38.8
	Female	588	61.2
Marital status	Single	181	18.8
	Married	550	57.2
	Widowed	97	10.1
	Divorced	133	13.8
Residence	Urban	681	70.9
	Rural	280	29.1
Religion	Orthodox	555	57.8
	Muslim	192	20.0
	Protestant	210	21.9
	other	3	0.3
Educational status	Illiterate	221	23.0
	Can read and write	60	6.2
	Primary cycle^&^	411	42.8
	Secondary school ^#^	206	21.4
	Diploma and above	63	6.6
Occupation	Housewife	227	23.6
	Farmer	145	15.1
	Governmental employee	110	11.4
	Non–govt. employee	36	3.7
	Merchant	215	22.4
	Laborer	157	16.3
	Others*	71	7.4
Family size category	≤ 5	905	94.2
	>5	56	5.8
Live with	Parent	173	18.0
	Relatives	57	5.9
	Spouse	48	5.0
	Spouse and children	503	52.3
	Alone	180	18.7
Source of drinking water	Tap	578	60.1
	Spring	251	26.1
	River	51	5.3
	Well	81	8.4
Family monthly income	<1,000 ETB^∧^	487	50.7
	1,001–2,000 ETB^∧^	346	36.0
	>2,001 ETB^∧^	128	13.3

Others^*^-students, police, drivers, miners, prisoners, privates.

Primary Cycle^&^= 1–8 grade.

Secondary school^#^= 9–12 grade.

ETB^∧^-Ethiopian Birr **(1 USD** **=** **52 ETB)**.

### Prevalence of undernutrition

**Figure 1:**
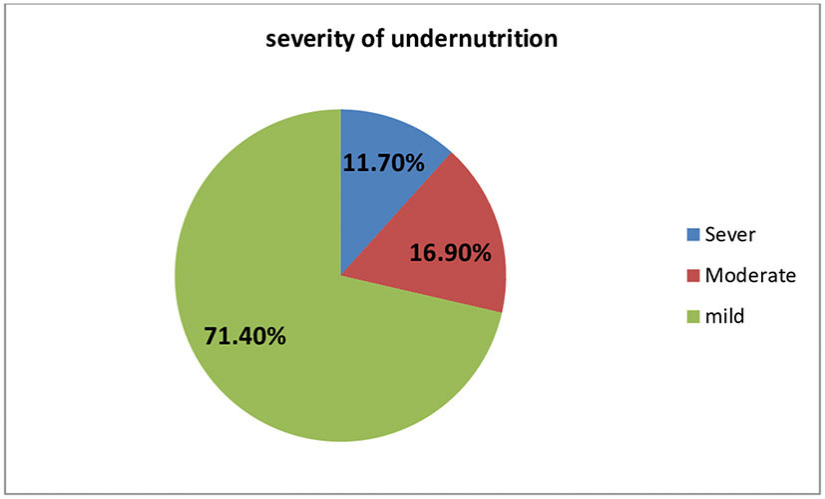
Severity of undernutrition in the study participants at Bench Maji Zone.

The total level of undernutrition among individuals on ART in this study was 16.0% (*n* = 154) (95% CI = 13.8–18.3). Among the 154 undernourished individuals, 11.7% were severe, 16.9% moderate, and 71.4% were mildly undernourished (Figure 1).

### Lifestyle of participants

Out of the total study participants, most of them (*n* = 887, 92.3%) did not smoke cigarettes and only 206 (21.4%) drank alcohol. Few study participants (*n* = 106, 11.0%) chewed khat and a majority of them (*n* = 692, 72.0) performed regular physical exercise (Table 2).

**Table 2:** Variables related to the lifestyle of the study participants at Bench Maji Zone, Ethiopia, 2018.

**Variable**	**Category**	**Frequency**	**Percent**
		**(*n* = 961)**	**(%)**
Smoking cigarette	Yes	74	7.7
	No	887	92.3
Status of smoking	Former smoker	59	79.7
	Current smoker	15	20.3
Consuming alcohol	Yes	206	21.4
	No	755	78.6
Frequency of taking alcohol	Daily	48	23.3
	Once per week	29	14.1
	Twice per week	47	22.8
	Three times per week	67	32.5
	Four times per week	13	6.3
	Other	2	1.0
Chewing khat^*^	Yes	106	11.0
	No	855	89.0
Regular physical exercise	Yes	269	28.0
	No	692	72.0

* Khat is a flowering plant native to the East and West Hararghe zones of Ethiopia. Khat contains the alkaloid cathinone, a stimulant, which is said to cause excitement, loss of appetite, and euphoria.

### Medical and health status of the participants

The majority of the respondents (*n* = 870, 90.5%) were at WHO clinical stage I of HIV during the previous 6 months. The CD4 number for most of the participants (*n* = 651, 67.7%) was >500 cell/mm^3^ and only 100 (10.4%) developed OIs in the previous 6 months. Approximately 329 (32%) had tuberculosis and 107 (11.1%) had other gastrointestinal symptoms. Most of the respondents (*n* = 633, 65.95%) spend >5 years since being diagnosed with HIV, and 693 (72.1%) spend >3 years since they started ART drugs. The most used HAART regimen for 597 (62.1%) participants was TDF+TC+EFV; most of whom (*n* = 899, 93.5%) showed good drug adherence, and only 47 (4.9%) developed side effects of HAART (Table 3).

**Table 3:** Variable related to the medical factor of the study participants at Bench Maji Zone, southwest, Ethiopia, 2018.

**Variable**	**Category**	**Frequency**	**Percent**
		**(*n* = 961)**	**(%)**
WHO Clinical stage of the	Stage-I	870	90.5
disease	Stage-II	34	3.5
	Stage-III and IV	57	5.9
Recent CD4 count of the	<200	64	6.7
patient	201–499	246	25.6
	>500	651	67.7
Previous Opportunistic	Yes	100	10.4
infection	No	861	89.6
Type of previous	Oral candidiasis	18	18.0
opportunistic infection the	Tuberculosis	32	32.0
patient developed **(*****n*** **=** **100)**	Chronic diarrhea	21	21.0
	Anemia	13	13.0
	Other^∧^	16	16.0
Other gastrointestinal	Yes	107	11.1
symptoms developed	No	854	88.9
Type gastrointestinal	Poor appetite/Anorexia	47	43.1
symptoms developed	Difficulty of eating	39	35.8
**(*****n*** **=** **109)**	Nausea and vomiting	23	21.1
	Other^#^	47	43.1
Time since HIV Diagnosis	<12 months	82	8.5
in months	13–36 months	134	13.9
	37–60 months	112	11.7
	>61 months	633	65.9
Current ART regimen	1c (AZT+3TC+NVP)	200	20.8
	1d (AZT+3TC+EFV)	90	9.4
	1e (TDF+TC+EFV)	597	62.1
	1f (TDF+3TC+NVP)	47	4.9
	1g (ABC+3TC+EFV)	4	0.4
	2b (TDF+3TC+LPV/r)	3	0.3
	2c (TDF+3TC+LPV/r)	2	0.2
	2e (AZT-3TC-LPV/r)	1	0.1
	2f (AZT-3TC-ATV/r)	5	0.5
	Other*	12	1.2
Duration of ART in month	<6 months	49	5.1
	7–12 months	67	7.0
	13–36months	152	15.8
	>37 months	693	72.1
Side effect of HAART	Yes	47	4.9
	No	914	95.1
Doses of ART drug	30 doses	404	42.0
	60 doses	275	28.6
	90 and 180 doses	282	29.3
Adherence status	Poor	44	4.6
	Fair	18	1.9
	Good	899	93.5

Other^*^- TDF+3TC+ATV/r, ABC+3TC+LPV/r, ABC+3TC+ATV/r.

Other^∧^-pneumonia, epilepsy, herpes zoster.

Other^#^ - PUD, Dyspepsia.

### Nutritional related factors

Among the respondents, only 64 (6.7%) received food aid, of which 49 (76.6%) received it from the government, with Plumpy'Nut the most common (*n* = 43, 68.3%) type of food. The majority of the respondents (*n* = 610, 63.5%) received dietary counseling and most of them, 884 (92.0%), had good food frequency (diet > 3 times per day) (Table 4).

**Table 4:** Nutritional-related factors of the study participants at Bench Maji Zone, Ethiopia, 2018.

**Variable**	**Category**	**Frequency**	**Percent**
		**(*n* = 961)**	**(%)**
Recipient of food aid	Yes	64	6.7
	No	897	93.3
From whom the patient	From government	49	5.1
receives food aid **(*****n*** **=** **64)**	Non-government	14	1.5
	Other*	1	0.1
Types of food ration the	Plum nut	43	68.3
patient receives **(*****n*** **=** **63)**	Oils and other cereals	10	15.9
	Other^$^	10	15.9
Dietary counseling	Yes	610	63.5
	No	351	36.5
Meal frequency	Poor food frequency	77	8.0
	Good food frequency	884	92.0

Other^$^-money, soap.

Other^*^-relatives.

### Diversified diet

A total dietary diversity score was calculated from a 24 h recalled list of food items (nine food groups) consumed over the previous day, which revealed that about half of the study participants 489 (50.9%) had a diversified diet.

### Factors affecting undernutrition among people on HAART

In multivariable logistic regression analysis, factors contributing to undernutrition were identified; age, marital status, GI symptoms, and WHO clinical stage were found to have a statistically significant association with undernutrition among people on HAART.

Patients on HAART aged ≥50 were two times more likely to develop undernutrition compared to persons of younger age [AOR 2.5, 95% CI (1.1–5.6)]. Likewise, single people were two times more likely to develop undernutrition compared to their married counterparts [AOR 2.2, 95% CI (1.4–3.7)]. Patients on HAART who manifested GI symptoms were three times more likely to face undernutrition compared to others who did not [AOR 2.6, 95% CI (1.5–4.4)]. Individuals on ART who were at WHO clinical stages III and IV were three times more likely to be undernourished compared to their counterparts [AOR 2.8, 95% CI (1.3–6.0)] (Table 5).

**Table 5:** Multivariable logistic regression analysis of undernutrition and its associated factors among people on HAART in health facilities in the Bench Maji Zone, Ethiopia, 2018.

**Variable**	**Category**	**Undernourished**		**COR^&^ (95% CI)**	**AOR^@^ (95% CI)**	***P*-value**
		**No**	**Yes**			
Age	18–29	267	53	1	**1**	
	30–39	354	58	0.8 (0.6–1.2)	1.2 (0.7–1.9)	0.554
	40–49	149	30	1.0 (0.6–1.7)	1.4 (0.8–2.5)	0.216
	≥50	37	13	1.8 (0.9–3.6)	2.5 (1.1–5.6)^*^	**0.028**
Marital status	Married	480	70	**1**	**1**	
	Single	136	45	2.3 (1.5–3.5)	2.2 (1.4–3.7)^*^	**0.001**
	Widowed	78	19	1.7 (1.0–2.9)	1.1 (0.6–2.0)	0.773
	Divorced	113	20	1.2 (0.7–2.1)	0.9 (0.5–1.5)	0.585
Residence	Urban	578	103	1	**1**	
	Rural	229	51	1.3 (0.9–1.8)	1.3 (0.8–2.0)	0.300
Educational status	Illiterate	223	58	1.8 (0.8–4.0)	1.6 (0.7–3.9)	0.271
	Primary (1–8)	349	62	1.2 (0.6–2.7)	1.2 (0.5–2.8)	0.675
	Secondary school (9–12)	180	26	1.0 (0.4–2.3)	0.9 (0.4–2.3)	0.896
	Diploma and above	55	8	1	1	
Source of drinking water	Tape water	487	91	1	**1**	
	Spring water	213	38	1.0 (0.6–1.4)	0.9 (0.6–1.5)	0.736
	Liver water	38	13	1.8 (0.9–3.6)	1.7 (0.8–3.8)	0.163
	Well water	69	12	0.9 (0.5–1.8)	0.9 (0.4–1.8)	0.733
CD4 count	≤ 200	45	19	2.3 (1.3–4.0)	1.5 (0.8–2.9)	0.268
	201–499	213	33	0.8 (0.5–1.3)	0.7 (0.4–1.1)	0.135
	≥500	549	102	1		
OI	Yes	69	31	2.7 (1.7–4.3)	1.0 (0.5–2.0)	.950
	No	738	123	1	1	
GI symptom	Yes	67	40	3.9 (2.5–6.0)	2.6 (1.5–4.4)^*^	**0.001**
	No	740	114	1	**1**	
WHO clinical stage	Stage one	745	125	1	1	
	Stage two	27	7	1.5 (0.7–3.6)	1.5 (0.6–3.7)	0.388
	Stage three and four	35	22	3.5 (1.9–6.4)	2.8 (1.3–6.0)^*^	**0.010**
Time since HIV diagnosis	<12 months	60	22	2.1 (1.2–3.6)	1.3 (0.3–5.3)	0.720
	13–36 months	112	22	1.1 (0.7–1.9)	0.9 (0.4–2.2)	0.822
	37–60 months	96	16	1.0 (0.5–1.7)	0.7 (0.4–1.4)	0.346
	≥61 months	539	94	1	1	
Current ART regimen	1c (AZT+3TC+NVP)	164	36	1	1	
	1d (AZT+3TC+EFV)	73	17	1.1 (0.6–2.0)	1.2 (0.6–2.3)	0.659
	1e (TDF+3TC+EFV)	504	93	0.8 (0.6–1.3)	0.7 (0.4–1.1)	0.106
	Other	66	8	0.6 (0.2–1.3)	0.5 (0.2–1.1)	0.078
Duration on ART	≤ 6 months	35	14	2.3 (1.2–4.5)	1.3 (0.3–5.6)	0.767
	7–12 months	55	12	1.3 (0.7–2.5)	1.0 (0.3–3.5)	0.975
	12–36 months	125	27	1.3 (0.8–2.0)	1.4 (0.6–3.1)	0.433
	>36 months	592	101	1	1	
Side effects of ART	Yes	34	13	2.1 (1.2–4.1)	1.4 (0.7–3.2)	0.361
	No	773	141	1	1	
Drug adherence	Poor	32	12	2.1 (1.0–4.1)	1.7 (0.8–3.8)	0.207
	Fair	14	4	1.6 (0.5–4.9)	1.4 (0.4–4.6)	0.563
	Good	761	138	1	1	
Food frequency ^$^	Poor	54	23	2.4 (1.5–4.1)	1.7 (0.9–3.0)	0.110
	Good	753	131	1		
Diet counseling	Yes	505	105	1	1	
	No	302	49	0.8 (0.5–1.1)	0.8 (0.5–1.2)	0.190

* Statistically significant, ^&^ = Crude Odds Ratio, ^@^ = Adjusted Odds Ratio.

$ Food frequency is poor if less 3 per day, and good if 3 and above per day. Bold values means statistically significant.

## Discussion

This study identified the level of undernutrition among adult people receiving Highly Active Anti-Retro Viral Therapy to be 16%. HIV/AIDS had a direct or indirect impact on the nutritional status of the patients receiving HAART. The level of undernutrition identified in this study was lower than in the studies conducted in different countries; 43% in Brazil (20), 19.5% in Tanzania (26), 19.2% in Senegal (27), and 19.9% in Nepal (28). Similarly, it was also lower than in other studies conducted in Ethiopia: Hosana (9), Nekemte Referral Hospital (10), Butajira (11), Dembia district (12), Wolaita Sodo (13), and Bahir Dar Felege Hiwot Referral Hospital (14), which were 31.2, 27, 25.2, 23.2, 26.6, and 25.5%, respectively. The discrepancy might be because of the study period and increased health-seeking behavior of the community from time to time, which enhances early detection and treatment of HIV. Another reason might be the difference in the socioeconomic status of the community, which has a direct relation to undernutrition and increased government interventions such as early initiation of HAART, and supplementation of diet.

The level of undernutrition found in this study was higher than that indicated in other studies carried out in Zimbabwe (21) and in Dilla University Referral Hospital of Ethiopia (24), which were 10% and 12.3%, respectively. The difference might be because of the study area and intervention on dietary practice.

In this study, some associated factors were identified. Among the sociodemographic factors, age and marital status were significantly associated with undernutrition in people on HAART. This finding is consistent with a study conducted in Brazil (20). It is known that as age increases immunity decreases, which results in different diseases and opportunistic infections (OI) that affect the appetite and food intake of individuals. In this study, being single was also associated with undernutrition. This may indicate that unmarried individuals may be at a risk to develop HIV infection, and if infected, OI may follow affecting food intake. The association between marital status and undernutrition depends on the social and economic status of the individuals. It might be also related to the psychological conditions of individuals, where married persons follow HIV treatments and care more diligently as they get the required support from their partners. The study carried out in the Dembia district of the Amhara region and Jimma Medical Center in Oromia, Ethiopia, indicated that widowed people were associated with undernutrition among people on HAART (12, 17).

The other factors associated with undernutrition among adult people on ART in this study were developing gastrointestinal symptoms during the last 6 months. This is consistent with the studies conducted in Felege Hiwot Referral Hospital (Bahir Dar, Ethiopia) that indicated eating difficulty as a predictor (14). Besides, a similar study conducted in Brazil and the Hararghe zone and Dilla University Referral Hospital in Ethiopia showed that gastrointestinal symptoms were associated factors of undernutrition among adult people taking HAART drugs (15, 20, 24). This might be because people with gastrointestinal symptoms do not consume food properly, and poor absorption of the food may also lead to undernutrition. In this study, a WHO clinical stage of AIDS disease was also significantly associated with people who were undernourished and on HAART drugs. This finding is similar to the study conducted in Ethiopia in 2015 at Hosanna town Nekemt Referral Hospital, and Dilla University Referral Hospital (9, 10, 24). It is also consistent with recently published studies conducted in other regions of Ethiopia and Zimbabwe (15–17, 19, 21, 22). It is known that in advanced WHO clinical stages III and IV of the AIDs disease, the patient develops many opportunistic infections that affect the appetite and it is the stage at which people are unable to eat.

The main strength of this study is that it was conducted on sufficient sample size. However, this study may have limitations such as an inter-observer (measurer) error during measurements and also recall bias in some information.

## Conclusion

From the findings of this study, it can be concluded that even though it is relatively lower than the studies conducted in some regions of Ethiopia, significant numbers of people on HAART drugs in the study area are still undernourished. This shows that HIV/AIDS has a direct or indirect impact on the nutritional status of the persons receiving HAART and indicates that health professionals should follow closely patients on treatment to identify those at a risk to develop malnutrition. Age, marital status, the WHO clinical stage of disease, and the development of gastrointestinal symptoms were identified as factors of undernutrition among adult people on HAART.

## Data availability statement

The original contributions presented in the study are included in the article/supplementary material, further inquiries can be directed to the corresponding author.

## Ethics statement

The studies involving human participants were reviewed and approved by the Mizan Tepi University research committee. Written informed consent for participation was not required for this study in accordance with the national legislation and the institutional requirements.

## Author contributions

TR designed, wrote the proposal, searched for funds, undertook the data analysis, drafted the manuscript, supervised the study, and ensured data quality. TG assisted in the analysis and interpretation of data, supervised the work, searched for resources, and edited the manuscript. All authors critically reviewed the manuscript. The corresponding author did the analysis, drafted the manuscript, and had the responsibility to submit the manuscript for publication. All authors have read and approved the manuscript.

## Funding

Mizan Tepi University funded the work of this research, but not for publication.

## Conflict of interest

The authors declare that the research was conducted in the absence of any commercial or financial relationships that could be construed as a potential conflict of interest.

## Publisher's note

All claims expressed in this article are solely those of the authors and do not necessarily represent those of their affiliated organizations, or those of the publisher, the editors and the reviewers. Any product that may be evaluated in this article, or claim that may be made by its manufacturer, is not guaranteed or endorsed by the publisher.

## Abbreviations

AIDS: Acquired Immunodeficiency Syndrome
ART: Antiretroviral Treatment
ARV: Antiretroviral
BMI: Body Mass Index
FMOH: Federal Ministry of Health
HAART: Highly Active Antiretroviral Therapy
HAPCO: HIV and AIDS Prevention and Control Office
HIV: Human Immuno-Deficiency Virus
MUAC: Measurement of upper Arm Circumferences
PPLHIV: People living with HIV/AIDS
RUTF: Ready to use therapeutic food.
